# Brain macrophage development, diversity and dysregulation in health and disease

**DOI:** 10.1038/s41423-023-01053-6

**Published:** 2023-06-26

**Authors:** Aymeric Silvin, Jiawen Qian, Florent Ginhoux

**Affiliations:** 1 INSERM U1015, Gustave Roussy Cancer Campus, Villejuif, 94800 France; 2 Shanghai Institute of Immunology, Department of Immunology and Microbiology, Shanghai Jiao Tong University School of Medicine, Shanghai, 200025 China; 3 Singapore Immunology Network, Agency for Science, Technology and Research, Singapore, 138648 Republic of Singapore; 4 Translational Immunology Institute, SingHealth Duke-NUS Academic Medical Centre, Singapore, 169856 Singapore

**Keywords:** Microglia, Macrophages, Meninges, Brain, Inflammation, Microglial cells, Neuroimmunology

## Abstract

Brain macrophages include microglia in the parenchyma, border-associated macrophages in the meningeal-choroid plexus-perivascular space, and monocyte-derived macrophages that infiltrate the brain under various disease conditions. The vast heterogeneity of these cells has been elucidated over the last decade using revolutionary multiomics technologies. As such, we can now start to define these various macrophage populations according to their ontogeny and their diverse functional programs during brain development, homeostasis and disease pathogenesis. In this review, we first outline the critical roles played by brain macrophages during development and healthy aging. We then discuss how brain macrophages might undergo reprogramming and contribute to neurodegenerative disorders, autoimmune diseases, and glioma. Finally, we speculate about the most recent and ongoing discoveries that are prompting translational attempts to leverage brain macrophages as prognostic markers or therapeutic targets for diseases that affect the brain.

## Introduction

Many studies on the origins, subtypes, and roles of brain macrophages have been conducted over the past decade. While our understanding of the origins, heterogeneity and functional states of these cells is still controversial in the field, data generated using single-cell technologies have led to a rapid increase in new theories and research directions related to how we can understand the complexity of these cells and how we might target these cells in health and disease.

Among brain macrophages, microglia have long been the most well-studied cell population.

Microglia are resident macrophages in the brain parenchyma and play crucial roles in brain development, homeostasis, and immune surveillance. Discovered in 1919 by Del Rio-Hortega, microglia have been studied for more than a century, and our understanding of these cells has increased. Key breakthroughs in microglia research include the discovery of their unique development from primitive macrophages, their local self-maintenance through proliferation and their ability to acquire unique functional states in response to changing environments during aging and disease conditions, especially with the description of disease-associated microglia (DAM) that arise during neurodegeneration.

**Fig. 1:**
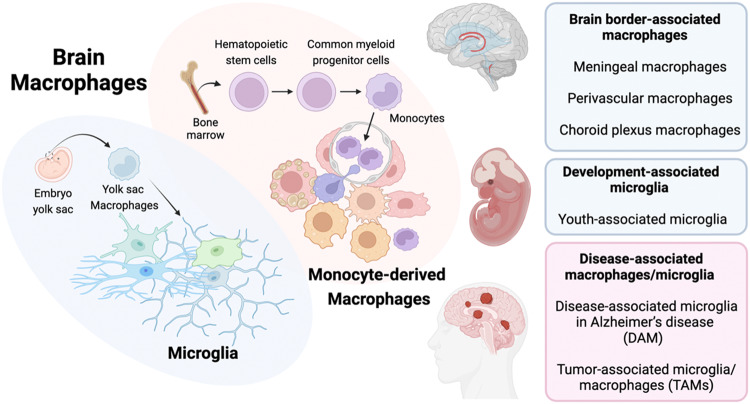
Classification of brain macrophages in health and disease

Innovative research on types of macrophages in the brain other than microglia started later, and this research was fueled by the notions that tissue macrophage populations differ according to their ontogeny (embryonic versus adult progenitors) and adapt to their niche of residence through specific molecular crosstalk with neighboring cells. As such, with the help of high-dimensional approaches, such as flow cytometry, mass cytometry and single-cell RNA sequencing (scRNA-seq), the precise phenotypes and transcriptomic programs of border-associated macrophages in the meningeal-choroid plexus-perivascular space were revealed. Our understanding and classification of macrophages has greatly improved, and the heterogeneity of the programs of the central nervous system has drastically increased. Due to the high plasticity of macrophages, their classification is complex and multifaceted (Fig. 1). Several subtypes of brain macrophages have been reported, from several microglial states to border-associated macrophage subtypes depending on their localizations to disease-associated microglial subtypes, in addition to monocyte-derived macrophages that infiltrate the brain under various disease conditions. These distinct cell populations exhibit functional and phenotypic differences, and they contribute to the maintenance of homeostasis, immune surveillance, and neuroinflammation regulation, playing critical roles in brain health and disease.

Findings from more than 30 years have highlighted the importance of integrating the ontogeny, location, disease programming, functional features, and gene expression patterns of brain macrophages to gain insights into their different functions and roles in disease. This is particularly important given that the treatment of brain disorders is hindered by the organ’s complexity, disease heterogeneity, and insufficient mechanistic knowledge. Investigating the role of brain macrophages in maintaining homeostasis or contributing to pathogenicity via neuroinflammation mechanisms could reveal novel therapeutic approaches, potentially enhancing the efficacy of treatments for various brain disorders. In this review, we discuss the roles of the various macrophage populations that arise during healthy embryonic development and adulthood. We discuss their function in contributing to brain development and briefly outline how they maintain this organ after birth. We then highlight the contributions of these cells to disorders of the brain, including neurodegenerative disorders (Alzheimer’s disease, Parkinson disease, and amyotrophic lateral sclerosis), autoimmune diseases (multiple sclerosis) and glioma, as well as the possible parameters that affect their functions that remain to be elucidated. Our hope is that increasing our knowledge of brain macrophage ontogeny and functions will render these cells suitable as prognostic, diagnostic or therapeutic targets.

### Macrophage populations involved in normal embryonic brain development

Primitive macrophages are the first immune cells to emerge from the yolk sac (YS) during early fetal development, and they colonize all tissue rudiments, including the brain [1–3]. These early macrophages contribute to organ and tissue functionalization by mediating various cellular processes, such as angiogenesis [4], neurogenesis [5], erythropoiesis [6], and cell debris clearance [7]. Primitive macrophages also engage immune signaling pathways that contribute to fetal development within the tightly regulated *in utero* space. These signaling pathways contribute to the immune protection of the organism after birth [8]. In the following sections, we outline the emergence and subsequent roles of the various macrophage populations that help mediate brain development.

#### Microglial development and function

**Fig. 2:**
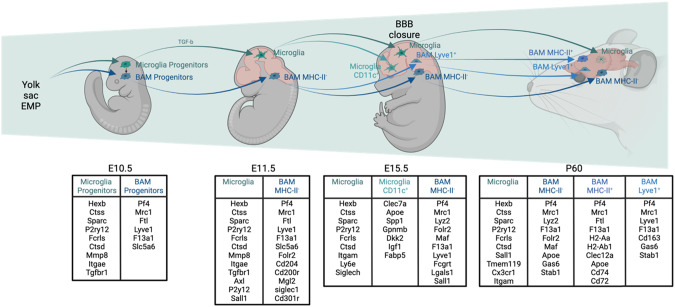
Macrophage heterogeneity during brain embryonic development and specific gene signatures

Microglia arise from primitive macrophages that are generated in the YS and derived from erythroid myeloid progenitors (EMPs) [9]. Primitive macrophages start to populate the neuroectoderm of the murine fetus through the blood circulation at embryonic day (E) 8.5 and remain the only glial cells until the prebirth fetal stage, when astrocytes and oligodendrocytes emerge (Fig. 2). Interestingly, microglia differ in males and females as a result of distinct developmental trajectories [10]. For example, in mice, the number and shape of microglia varies in distinct brain areas between sexes [11, 12], likely due to the different number of X chromosomes [13], testosterone production at E16-18 in male fetuses [14] and responses to fetal gut colonization with the mother’s microbiota during embryogenesis [15]. Understanding more about this gut–brain axis is expected to shed light on why susceptibility to some brain diseases varies between males and females. During brain development, microglia play crucial roles in processes such as axon outgrowth and fasciculation, synapse pruning and neuron population regulation, and cell death and apoptosis via the secretion of apoptotic factors in a complement-dependent manner, and they play roles in survival through IGF-1 secretion [5, 15–17]. Further work is needed to understand precisely how fetal microglia contribute to embryonic brain development and how their dysfunction might contribute to disease.

#### Border-associated macrophage development and function

Border-associated macrophages (BAMs) are found at brain border structures and are important in regulating the exchange of molecules between the brain and peripheral tissues. The recent emergence of scRNA-seq techniques has helped us to elucidate the level of BAM heterogeneity in great detail [18]. We now know that BAMs include several types of macrophages, such as meningeal macrophages, perivascular macrophages (PVMs), and choroid plexus macrophages [18–21]. In turn, we have learned more about BAM ontogeny.

An initial study reported the presence of CD45^high^ macrophages in the meninges, choroid plexus or perivascular space [22], and the authors showed that as microglia, these central nervous system-associated macrophages (CAMs) were mainly of embryonic origin using fate mapping mouse models [22]. The same team later published a study conducted with high-throughput scRNA-seq coupled with intravital microscopy and ontogeny to highlight the ability of CAMs, also called BAMs, to self-renew and contribute to inflammation. They showed that PVMs maintained their self-renewal more than meningeal macrophages, suggesting a higher contribution to monocyte recruitment by meningeal macrophages in the context of inflammation [23]. Utz et al. found that BAMs are of embryonic origin and come from a distinct macrophage lineage of TGF-β-independent BAMs already present in the yolk sac [20]. In contrast, microglia are TGF-β dependent. Moreover, Silvin et al. showed that BAMs are renewed through monocyte recruitment in adulthood and upon aging [24]. Van Hove et al. in their single-cell atlas of brain macrophages, established and described several choroid plexus macrophage subpopulations in addition to differentiating dural and subdural BAMs [18].

BAMs are identified based on *Mrc1* gene/CD206 protein expression (Fig. 2). Meningeal macrophages can be further subdivided into two subpopulations based on MHC-II expression. Specifically, embryonic and postnatal meningeal macrophages are mostly CD206^+^MHC-II^-^; CD206^+^MHC-II^+^ meningeal macrophages emerge during childhood (Fig. 2) [18, 20, 24]. The reasons and mechanisms underlying this shift are unknown. Another subgroup of BAMs is named perivascular macrophages (PVMs) or choroid plexus macrophages. Recently, Brioschi et al. highlighted the fact that BAMs do not depend on SMAD4, while microglia do. Using Crybb1^cre^Smad4^Flox^ mice, the authors showed an arrest in microglial development, while BAM development was conserved [25]. The authors then discussed the origin of the different BAMs based on MHC-II and CD38 expression. While MHC-II has been previously reported by several articles to discriminate 2 subgroups of BAM similar to *Lyve1*, *Cd163* and *F13a1*, the authors suggest that MHC-II^+^ BAMs have a higher monocytic contributions than MHC-II^-^ BAMs [25].

### Macrophage populations involved in normal brain maturation and maintenance after birth

The brain undergoes massive reorganization after birth, which is accompanied by the emergence of other glial cells, including astrocytes and oligodendrocytes [26]. Many of the functions once performed by embryonic microglia are then performed by these glial cells. This implies that there is a change in microglial functions that helps to mature neuronal activities. Interestingly, microglial global RNA expression drastically increases after birth, suggesting strong microglial maturation at this stage. The factors involved in this increase in microglial RNA expression remain poorly understood. Below, we outline the contributions of macrophages to brain maturation and the integrity of the blood‒brain barrier (BBB) after birth.

#### Microglial diversification

Researchers have observed the postbirth emergence of a unique microglia subpopulation that is defined by CD11c (Itgax) expression and is enriched in the corpus callosum, and these researchers revealed the unique transcriptomic signature of this population using bulk RNA-seq; this population contributes to proper oligodendrocyte development and homeostasis, supporting brain myelination [27, 28]. These CD11c^+^ microglia might play a role in myelogenesis. Hammond et al. described a similar cell population by scRNA-seq level and, due to their expression of *Itgax* and localization along the axons, called them axon track microglia (ATMs). These cells seemed to appear after birth but disappeared by postnatal day (P) 14 in mice. Silvin et al. (2022) found that CD11c^+^ microglia and ATMs are the same cell population [24] that appear in the rudimentary murine brain at E14 (Fig. 2). These cells express a specific gene signature comprising *Apoe*, *Gpnmb*, *Spp1*, *Igf1*, *Clec7a*, and *Itgax* and overexpress genes implicated in fatty acid beta oxidation, ketogenesis, the tricarboxylic acid cycle or glutathione redox reactions, which needs to be better understood [24]. Because these cells emerge during embryogenesis and remain for just a few weeks after birth, Silvin et al. renamed them youth-associated microglia (YAMs). Further work is now needed to better define the role of these cells in brain maturation.

Data from Erny et al. also suggest that the gut microbiota helps to maintain microglia maturation and function after birth [29]. Although the underlying mechanisms are only partially described, it seems that acetate production by the microbiota helps to ensure the metabolic fitness of microglia [30], acting at the epigenetic level through H3K4 methylation and H3K9 acetylation. This process is a key example of how metabolic support from cells or microorganisms contributes to cellular differentiation and function acquisition. Finally, while the role of the gut microbiota in mediating BAM maturation and function is unclear, studies in this area could shed crucial light on the dynamics of the blood‒brain barrier (BBB).

Indeed, gut microbiota play a known role in establishing another barrier, the blood–testis barrier (BTB) [31]. Even though these barriers differ in structure, the establishment of the blood brain barrier (BBB) and BTB is delayed in germ-free (GF) mice [32, 33], highlighting potential common factors that could be involved in the formation of both barriers. Depletion of gonadal testosterone in rats also in the permeability of the BBB [34]. The fact that CD206^+^MHC-II^+^ meningeal macrophages appear after birth could be a consequence of the increased microbiota complexity/diversity after birth and could contribute to the emergence of CD206^+^MHC-II^+^ BAMs, but these hypotheses remain to be proven (Fig. 2).

#### Meningeal macrophages

The BBB comprises three layers: the dura mater, arachnoid mater and pia mater. The BBB is highly vascularized [35] and surrounded by meningeal and PVMs that coexist in this space. Meningeal macrophages have a higher monocytic contribution than other brain macrophages but are mostly of embryonic origin after birth until 2 months of age (less than 10%) [24]. They play a key role in the control of viral infection [36], as exemplified during lymphocytic chroriomeningitis virus (LCMV) infection, where meningeal macrophages become infected and restrain viral infection to the meninges through an interferon response [36]. If meningeal macrophages are depleted (particularly CD206^+^MHC-II^+^ subtypes), LCMV infection propagates to the brain parenchyma and causes death.

Meningeal macrophages present antigens through MHC-II. This function is evidenced during experimental allergic encephalomyelitis, where these macrophages actively contribute to the clinical symptoms [37]. Other roles for meningeal macrophages include facilitating learning and memory [38]. Taken together, it seems that there are increasing numbers of roles for meningeal macrophages in several processes that maintain brain homeostasis, ranging from immune protection of the brain to cognition. However, it is crucial to characterize these cells by transcriptomic and spatial transcriptomic analyses to better understand their role and functional states.

#### Perivascular macrophages

The current consensus is that brain PVMs, which are characterized by the expression of *Cd206* and *Lyve1*, are probably of embryonic origin. PVMs contribute to BBB function by limiting the passage of molecules > 10 kDa [39]. These cells contribute to neutrophil recruitment, as shown by their depletion using clodronate during bacterial meningitis infection [40] and viral infection [41]. They also contribute to hypertensive pathogenesis, and PVMs recognize angiotensin II and produce ROS through NOX2 activation [42, 43]. A scRNA-seq analysis of brain PVMs in germ-free (GF) and specific pathogen-free (SPF) mice revealed specific gene signatures associated with the presence or absence of microbiota [44]. In contrast, no major changes were observed between meningeal macrophages in GF or SPF mice [44], suggesting that embryonically derived meningeal macrophages are not modified by the microbiota. However, a study of LPS-conditioned monocytes demonstrated a protective role at the meninges during ischemic brain injury, as evidenced by reduced expression of proinflammatory genes that are involved in neutrophil activation and decreased secretion of the chemotactic factor CSF3 [45, 46]. Interestingly, a model of acute ischemic stroke helped in understanding the role of acute monocyte recruitment in the brain and the role of monocyte-derived macrophages in the brain [47]. While these monocyte-derived macrophages strongly contribute to the first phase of postischemic stroke to reduce brain edema, BBB damage, neuronal apoptosis and cerebral ischemic infarction [48–51], they also seem to promote inflammation via reactive oxygen species, glutamate and chemokines in a second phase, causing secondary damage [52]. Recently, a study showed their involvement in Alzheimer’s disease [53], which will be discussed later. Thus, monocyte-derived meningeal macrophages and brain PVMs can be affected by the microbiota. It seems prudent, therefore, to keep in mind that with aging, microbiota dysbiosis and inflammaging monocytes, the balance between embryonically derived and monocyte-derived brain macrophages changes and that this effect could contribute to disease emergence.

### Interplay between macrophages, aging and inflammation

While our understanding of the intricacies of brain macrophage heterogeneity during homeostasis has drastically improved over recent years, our corresponding understanding in the context of aging and neurodegenerative diseases is lagging behind. However, improving our understanding of pathological mechanisms in which brain macrophages are involved will help us to define new therapeutic approaches. In the following sections, we outline the latest updates in understanding how macrophage origins and function change with aging.

#### Aging and senescence

**Fig. 3:**
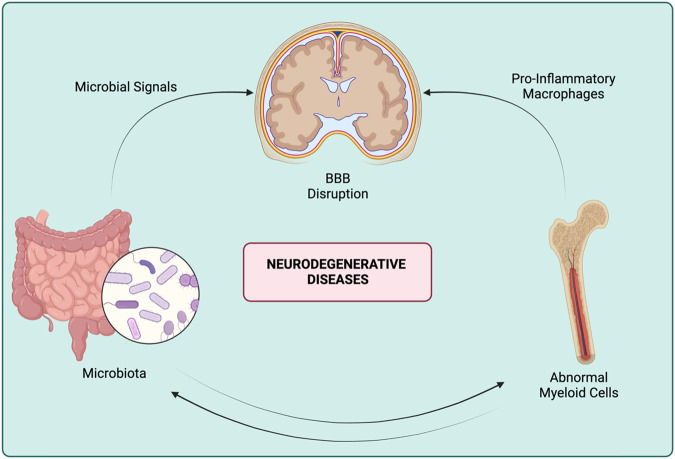
Neurodegenerative diseases that arise due to deterioration of barriers (gut and BBB) to inflammaging could impact brain macrophage populations that range from BAMs to microglia or monocyte-derived macrophages

The aging process is characterized by the deterioration of barriers, namely, deterioration of skin and leakiness of the gut mucosa and the BBB (Fig. 3). Aging affects every organ in the body, perhaps in part due to an encoded blood-based signature [54] but also as a result of processes including DNA damage, epigenetic changes, immune dysregulation, protein homeostasis and lysosomal dysfunction. Indeed, circulating factors such as CCL11, B2M, and IGF1 can modulate or even rejuvenate some organs [55–57]. The aging brain is characterized by the presence of abnormal neuronal lysosomes that promote the accumulation of macromolecules and subsequent neuronal cell dysfunction [58]. The resulting increase in dying cells and debris suggests that microglia might quickly accumulate such debris, as shown for myelin in aging microglia [59]. It was also shown that microglial transcription patterns differ depending on the brain region in which they reside, and this localization also induces a regional selective acquisition of an aging transcriptional signature [60]. Functionally, it was shown that lipid droplet accumulating microglia (LDAMs) emerge with aging [61]. These microglia are characterized by defective phagocytosis, the production of high levels of reactive oxygen species and the secretion of proinflammatory cytokines. These results suggest that with aging, microglia undergo transcriptional, functional and metabolic shifts that differ depending on the brain region [60–62]. Of course, it is currently difficult to determine whether such observations are the consequence or the cause of aging. Regardless, the drastic change in metabolic interplay between neurons and microglia due to lysosomal dysfunction and phagocytosis overload should be noted.

Hammond et al. provided a better understanding of brain macrophage heterogeneity in aging mice by comparing macrophage heterogeneity from embryonic time points to aging time points by scRNA-seq [63]. Consensus, however, is lacking regarding which macrophage signatures are indeed common among several studies. Silvin et al. used M-Verse to integrate several scRNA-seq datasets to construct a brain macrophage map in aging and neurodegenerative diseases [24]. They found that microglia change their signatures during aging and that monocyte-derived macrophages emerge and are characterized by an inflammatory signature; these macrophages are named disease inflammatory macrophages (DIMs).

#### Impact of aging on brain macrophage renewal

As discussed, several studies have shown that microglia and BAMs are of embryonic origin [18, 20, 25]. With aging, however, the proportion of these cells that are derived from monocytes increases in the brain [24]. Indeed, in the case of macrophage death, these cells are sometimes replaced by neighboring macrophages or by recruited monocytes. These results highlight the fact that brain macrophages in the aging brain also include monocyte-derived macrophages that were originally nearly absent from the pre- and postnatal brain parenchyma. These monocyte-derived macrophages that accumulate with age appear to be more prone to induce inflammation than their counterparts of embryonic origin (Fig. 3), as shown by Sierra et al. and Johnston et al. [64, 65]. While monocyte recruitment has been shown to contribute to wounding in brain lesions [66], we must keep in mind that aging directly impacts the anti-inflammatory properties of monocytes [67]. Interestingly, the circulating cytokines CCL11 and B2M could be part of a low-grade inflammation known as inflammaging and associated with monocytic cells [68]. While monocyte-derived macrophages can actively contribute to brain healing and homeostasis during adulthood, they may play the opposite role in aging. The proportion of monocyte-derived macrophages in the brain could, therefore, contribute to inflammaging.

Circulating monocytes in aged organisms are not similar to regular monocytes found in healthy, young adults [67]. For example, hippocampal macrophage gene expression drastically differs between mice aged 22 months and mice aged 4 months [60]. More studies are needed to determine whether this change in gene expression is associated with monocyte recruitment in this particular brain area. Such differences in gene expression might underlie observations such as worsened immunity against *Streptococcus pneumoniae* in aged mice at the systemic level, perhaps due to elevated TNFα produced by monocytes [69]. Indeed, this concept aligns with the fact that with aging, myelopoiesis is disturbed, and monocytes and neutrophils egress from the bone marrow too early [69]. Studies have already shown that aging monocytes display epigenetic alterations, particularly at the level of H3K27M [70], and clonal mutations of key genes such as TET2 or DNMT3A greatly impact myelopoiesis, as shown by Lim et al. or Assmus et al. [71, 72]. Knowing which factors contribute to the emergence of inflammaging monocytes and at which level these monocytes with a higher ability to drive inflammation during aging are impacted will no doubt help us to control systemic inflammaging and its impact on the brain.

#### Impact of aging on BBB integrity

Limits to the currently available molecular tool kit have rendered it difficult to study the BBB [73] and, in particular, the implications of aged BAMs on BBB integrity. Data from several studies have, however, shown that certain areas of the BBB are more sensitive to disruption than others [74, 75]. For example, a contrast-enhanced MRI protocol revealed that the BBB surrounding the human hippocampus seems more susceptible to breakdown than that surrounding the rest of the brain [74]. Macrophage activation has been associated with altered tight junction integrity and subsequent BBB permeability [76]. Monocyte-derived BAMs also accumulate in the murine BBB with age, as shown by Silvin et al. As discussed earlier, these BAMs could have an enhanced capacity to elicit inflammation at the level of the meninges and disrupt their integrity. Studies are now needed to understand the impact of these cells in the different specific brain regions as well as their contribution to aging and senescence.

### The contributions of macrophages to neurodegenerative disorders

With the aging population, the proportion of patients with neurodegenerative diseases such as Alzheimer’s or Parkinson’s disease is increasing worldwide. Pathological factors underlying these disorders are being identified, including dysregulation of macrophage functions that are essential for the homeostasis of cerebral tissue. In the last 15 years, many risk factors for neurodegenerative diseases have been identified. Some of these factors are closely associated with brain macrophages, such as TREM2 or APOE [77, 78]. These macrophage-associated risk factors highlight the importance of macrophages/microglia in the pathogenicity of neurodegenerative disease and suggest that neurodegenerative disease could be a consequence of microglial/BAM dysregulation.

#### Macrophage heterogeneity in Alzheimer’s disease and Parkinson disease

While Alzheimer’s and Parkinson’s diseases are two different pathologies that affect the brain, a common factor between them is immuno-inflammation, which causes toxic neuronal activation, neuronal death and the emergence of aggregates. These aggregates include amyloid beta and/or tau aggregates in Alzheimer’s disease [79, 80] and Lewy bodies in Parkinson’s disease [81]. The contribution of immuno-inflammation versus aggregates to disease symptoms is unclear and may be interrelated. Importantly, recent clinical data suggest that at least for Alzheimer’s disease, anti-TNFα antibodies might be protective [82]. Improving our understanding of the macrophage populations and related mechanisms that contribute to inflammation in this context is now urgently needed.

The development of scRNA-seq technologies has markedly facilitated studies on the heterogeneity of brain macrophages in Alzheimer’s disease [18, 83] and Parkinson’s disease. For example, using scRNA-seq, preliminary data from Schonhoff et al. suggest that BAMs are the predominant macrophage population contributing to inflammation in Parkinson’s disease [84]. They also showed that monocyte recruitment is decreased if inflammatory BAMs are depleted. Others have performed scRNA-seq integration of multiple datasets to also show that in both diseases, monocytes are recruited in higher numbers compared to regular aging. Specifically, in the case of Alzheimer’s disease, the recruited cells are characterized by inflammatory cytokine gene expression, including *Il6*, *Tnfa*, and *Il1b*, and thus are called disease inflammatory macrophages (DIMs) [24]. Another macrophage population described in Alzheimer’s disease is disease-associated microglia (DAM) [83]. These cells are dependent on TREM2 [83] and seem to reacquire a fetal-like program observed in CD11c+ embryonic microglia. These cells express very similar gene signatures, namely, *Spp1*, *Igf1*, *Gpnmb*, and *Dkk2*, and they have the ability to phagocytose amyloid beta aggregates and activate immunoregulatory pathways [18, 24, 83]. While originating from embryonic macrophages [24], the cues that regulate their emergence during neurodegenerative diseases remain unclear. Keren-Shaul et al. showed that the absence of TREM2 decreases DAM numbers and worsens Alzheimer’s disease symptoms in mice. These results suggest that TREM2 mutations could particularly impact this microglial subset. Recently, the potential role of perivascular macrophage-microglia interactions in Alzheimer’s disease was highlighted by De Schepper et al., revealing how PVM-derived SPP1 affects the ability of microglia to engulf synapses during the early stage of Alzheimer’s disease [53].

DAM and DIMs share a common gene expression profile, characterized by *Apoe* and *Trem2* expression [24]. DAMs, however, are of embryonic origin, depend on TREM2 to appear in the brain, and seem to be protective, while DIMs are monocyte-derived, express but are not dependent on TREM2 to appear in the brain, and contribute to inflammation [24]. As new populations of macrophages are identified and associated with these neurodegenerative diseases, work is needed to determine their precise contribution to pathogenesis.

#### BBB integrity in Alzheimer’s disease and Parkinson disease

The status of BBB integrity in neurodegenerative disorders is crucial to determine the possible route of drug actions. In the case of Alzheimer’s disease and Parkinson’s disease, the integrity of the BBB seems to be compromised by ongoing inflammation in the brain [85–89]. The BBB must be considered when developing innovative treatment strategies for these conditions, and understanding the role of BAMs in the maintenance of BBB integrity, as well as understanding whether the loss of BBB integrity impacts BAM or microglial functions, is crucial to propose innovative anti-neurodegenerative treatments (Fig. 3).

#### Gut and neurodegeneration

In recent years, the effect of the gut in the context of Parkinson disease and Alzheimer’s disease has been studied [90] (Fig. 3). While the role of the gut microbiota in Alzheimer’s disease remains to be elucidated, it might influence inflammatory responses in different locations, including in the bone marrow during the genesis of aged monocytes or in the brain by activating brain macrophages. Gut dysbiosis has been observed in patients with Alzheimer’s disease [91] and might influence Alzheimer’s disease progression via improved access of neurotoxins and bacterial compounds to the brain parenchyma as a result of a compromised BBB [92, 93]. In the case of Parkinson’s disease, a role of gut microbiota in controlling and regulating motor deficits and neuroinflammation has been reported [94]. Specifically, researchers showed that after fecal transplantation from patients with Parkinson’s disease into a Parkinson mouse model, the mice displayed enhanced physical impairment compared to those that received fecal transplantation from healthy human volunteers [94]. Taken together, it seems crucial to consider the microbiota, BBB integrity, and bone marrow myeloid cell aging as well as the impact of these factors on brain macrophages (BAMs, microglia, monocyte-derived macrophages or DAMs) to characterize the roles of these cells in these diseases.

### The contributions of macrophages to other neuroinflammatory disorders

Multiple sclerosis (MS) is a chronic autoimmune disease characterized by demyelination, gliosis, neurodegeneration in the CNS and inflammation [95]. Although it is not an autoimmune disease, amyotrophic lateral sclerosis (ALS) also results in similar degeneration but specifically in motor neurons. Neuroinflammation is a common feature of these two diseases [96, 97]. In MS, neuroinflammation is caused by autoreactive T cells, whereas in ALS, it occurs due to dysfunctional T regulatory cells; however, it has been reported that macrophages also contribute to disease pathophysiology. Indeed, MS and ALS are characterized by the recruitment of high numbers of monocytes to MS or ALS lesions, leading to the enrichment of monocyte-derived macrophages [98–100]. In the following sections, we discuss the precise roles of macrophages in these disorders.

#### Macrophage functions in MS and ALS

**Fig. 4:**
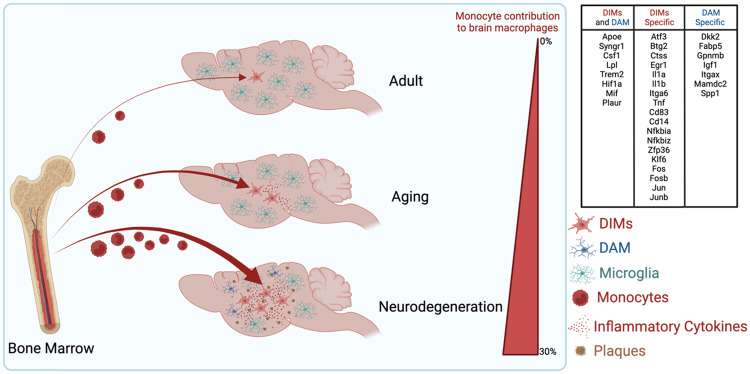
Disease-associated macrophage programs and their common versus specific gene signatures

Several studies have leveraged scRNA-seq techniques to map macrophage heterogeneity in MS and ALS. Keren-Shaul et al. defined a DAM signature based on *Apoe*, *Trem2*, *Lpl*, and *Ctsd* expression. Several studies in neurodegenerative diseases have reported cells expressing this signature at the scRNA level [23, 83, 101–103]. However, Silvin et al. argued that this signature was not specific enough and could also include DIMs [24]. Similarly, a population of cells previously identified as DAM in ALS by Keren-Shaul et al. (2017) could also include DIMs. Thus, a high level of precision is required to characterize macrophage signatures and accurately understand corresponding disease mechanisms. The DAM-specific signature was characterized by the unique overexpression of *Spp1*, *Dkk2*, *Fabp5*, *Gpnmb*, *Igf1*, *Itgax*, and *Mamdc2*, while the DIM signature was characterized by the overexpression of *Atf3*, *Btg2*, *Ccl4*, *Ctss*, *Dusp1*, *Egr1*, *Fos*, *Icam1*, *Ier2*, *Ier5*, *Il1a*, *Il1b*, *Itga6*, *Jun*, *Junb*, *Klf6*, *Tnf*, *Zfp36*, *Cd83*, *Fosb*, *Cd14*, *Fth1*, *Nfkbia*, and *Nfkbiz* [24]. The hallmark genes *Apoe*, *Trem2*, *Ctsd* and *Lpl* were not accurate enough to distinguish these two different macrophage populations (Fig. 4). MS lesions are also characterized by the presence of foamy macrophages containing abundant lipid droplets [104]; this disrupted lipophagy contributes to the inflammatory phenotype. Toxic lipids have also been identified in ALS or other neurodegenerative diseases [105].

#### BBB integrity in MS and ALS

BBB leakage has been widely reported in both MS and ALS [106–108]. In MS patients, beta interferon and methyl prednisolone can restore the integrity of the BBB [109, 110]. It is crucial to characterize the impact of these treatments on the functions of BAMs as well as other cell types to better understand their contribution to neurodegenerative diseases. Whether BBB leakage is homogeneous or restricted to particular areas in MS and ALS is unknown. For a long time, neurodegenerative diseases were considered brain-restricted conditions that were characterized by the formation of aggregates in areas that drug treatments could not reach; however, these dogmas are now being challenged as a result of data obtained using new technologies. The hypothesis of neuroinflammation was underestimated for a long time but now might be a promising treatment avenue. While much work remains to be conducted in order to characterize how various cell types function and interact in these diseases, early data suggest that macrophages are involved in the emergence of symptoms and subsequent disease development.

### Interactions between glioma and macrophages

Glioma is the most prevalent primary tumor of the brain parenchyma. Medulloblastoma and pediatric midline gliomas occur in early childhood, while glioblastoma (GBM) and anaplastic astrocytoma are more common during late adulthood and are associated with aging [111, 112]. Sex-related factors are thought to be involved in the pathogenesis of glioma [113, 114]. Specifically, it has been observed that the incidence of GBM is higher in males than in females, with a male-to-female ratio usually ranging from 1.5:1 to 2:1 [115, 116]. In addition, investigations with patients with GBM undergoing standard-of-care treatment have revealed that females display a markedly superior survival time compared to their male counterparts [117]. Numerous primary and metastatic tumors are infiltrated to varying degrees by tumor-associated macrophages (TAMs) — a mixed population of cells with different ontogeny. At the population level, TAMs participate in multiple biological processes of tumorigenesis, including tumor growth, tumor invasion, tumor angiogenesis and immune evasion [118, 119]. Developments in single-cell omics and fate mapping systems have, however, made it feasible to separately investigate the microglia and monocyte-derived macrophages (MDMs) comprising TAMs and thus elucidate their distinct roles in glioma pathogenesis. In the following sections, we focus on the spatial distribution and functional characteristics of these brain macrophages in glioma.

#### Macrophage abundance in glioma

High-grade gliomas such as GBM tend to contain a higher abundance of TAMs than low-grade gliomas such as oligodendroglioma [120]. Moreover, an scRNA-seq analysis revealed that recurrent GBM has more infiltrating TAMs than primary GBM [121]. The specific molecular subtype of glioma can affect the abundance and functional characteristics of macrophages. For example, greater macrophage enrichment was observed in the mesenchymal subtype of high-grade glioma than in the proneural and classical subtypes [122, 123]. This difference could be due to the NF1 mutation typically found in mesenchymal GBM, as NF1 can regulate myeloid cell chemotaxis [123]. Mutations in isocitrate dehydrogenase (IDH) genes also affect macrophage infiltration. Specifically, an scRNA-seq analysis of IDH-WT and IDH-mutant GBM samples showed that IDH-WT GBM has a higher level of infiltrating macrophages [124]. This finding might explain, in part, the better prognosis observed in patients with IDH-mutant versus IDH-WT gliomas.

#### TAM ontogeny in glioma

TAMs are composed of ontogenically distinct populations, given that this population comprises both microglia-derived TAMs (TAM-MG) and MDM-derived TAMs (TAM-MDMs). The development of fate-mapping models, such as Cx3cr1-CreER mice, now makes it possible to distinguish these two populations in mice with glioma [125]. For example, Bowman et al. utilized this tool to establish an orthotopic, syngeneic GL261 glioma model and then revealed the transcriptomic differences between these two TAM populations by bulk RNA sequencing [126]. Others have subsequently investigated the TAM-MG and TAM-MDM subpopulations by scRNA-seq [121, 127]. Now, functional validation of these cells is required to understand the impact of these genetic differences.

#### Spatial heterogeneity of microglia and MDMs in glioma

**Fig. 5:**
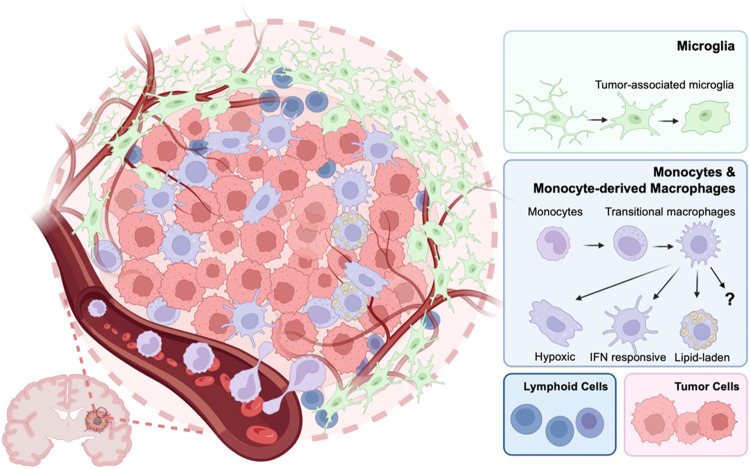
Distribution of microglia and monocyte-derived macrophages in glioma

TAM-MG and TAM-MDMs exhibited different spatial distributions in glioma (Fig. 5). After an scRNA-seq analysis of microdissected human glioma samples, Darmanis et al. found that infiltrating TAM-MDMs predominantly distribute to the tumor core, while TAM-MGs distribute in the tumor periphery [128]. Others have verified these findings [120, 129], including in a mouse glioma model using a fate-mapping system [121]. Others have revealed that TAM-MDMs are recruited during the early stage of glioblastoma tumorigenesis and are mainly located in perivascular regions [130]. It might be the case, therefore, that the distribution of these two ontogenically distinct cell populations varies with tumor type, location, and other factors.

The differential distribution of TAM-MGs and TAM-MDMs within tumors can have functional consequences, such as in the interaction between TAMs and T cells. Although MHC-II expression could be induced in both populations, and the TAM-MDMs present higher expression of MHC-II than TAM-MGs [121, 126, 127], these TAM-MDMs cannot interact with T cells localized in the tumor periphery [131]. Therefore, the differential distribution might explain why TAM-MDMs fail to process and present antigens to T cells in the glioma microenvironment and thus account for the diminished antitumor immune response of TAM-MDMs. In contrast, microglia colocalize with T cells in the tumor periphery [131]; thus, MHC-II+ TAMs-MG might partially activate T cells [132]. Further research is needed to disentangle whether the functional differences between these two populations occur due to their intrinsic distinct ontogeny, their distribution in the glioma microenvironment, or both.

#### Distinguishing microglia and MDMs in glioma

Research is needed to identify reliable and stable markers that can distinguish TAM-MGs from TAM-MDMs in glioma to better delineate their role in tumorigenesis. CD45 expression levels were originally considered a suitable marker to distinguish these two cell populations: CD11b+CD45high cells were identified as macrophages, and CD11b+CD45low cells were identified as microglia [133]. However, studies using a GL261 mouse model identified CD45 upregulation in the microglial population [134, 135]. Later work comparing steady-state microglia and macrophages revealed that CX3CR1 and CCR2 might be candidate markers to distinguish these cell populations, but these markers proved unreliable in the context of glioma. Specifically, MDM markers, including Ly6C and CCR2, were downregulated during MDM activation or differentiation in the glioma microenvironment [136]. These issues have subsequently led to the generation of conflicting results between studies [126, 134].

More recently, P2RY12 and CD49d were proposed to be robust markers for TAM-MGs and TAM-MDMs, respectively, after lineage-tracing studies were conducted in mice and validation studies were conducted with human glioma specimens [126, 129, 134]. Altered expression of these widely used microglial markers was subsequently observed during the course of CNS diseases. P2RY12 serves as a prototypical example of altered microglial markers during brain disorders, and a decrease in its expression is a prominent feature of microglia in Alzheimer’s disease [137] and glioma [138]. Other markers for microglia in homeostatic conditions, such as *Cx3cr1*, *Sall1*, and *Tmem119*, are also downregulated in glioma [138]. Despite some incremental progress, a lack of widely accepted cell markers limits the functional confirmation of these two cell populations.

#### MDM reprogramming in the tumor microenvironment

MDMs are recruited to glioma during tumorigenesis and represent one-third of the infiltrating myeloid cells in the tumor microenvironment at the late stage [126]. Once recruited, these TAM-MDMs undergo reprogramming to help tumor evade the immune system. Data derived from a GL261 glioma model indicated that TAM-MDM precursors are Ly6C+ classical monocytes [121]. When recruited to the tumor environment, these cells further differentiate into TAM-MDMs with varying phenotypes depending on the local niche. For example, Antunes et al. conducted a detailed and delicate analysis of the various MDM subpopulations in murine and human glioma samples and identified IFN-responsive, *Sepp1*+, hypoxic, and phagocytic subpopulations [121].

While functional validation is ongoing to verify the role of these MDM subsets in glioma progression, we are already gaining some insight into MDM reprogramming. For example, Bowman et al. reported that compared with TAM-MGs, TAM-MDMs were enriched in immunosuppressive cytokines such as IL-10 and chemokines that contribute to wound healing such as *Ccl22*, *Ccl17*, *Cxcl2* and *Cxcl3* [126]. Friedrich et al. demonstrated that disrupted tryptophan metabolism in IDH-mutant glioma hindered the differentiation of myeloid cells and caused the upregulation of IL-10 and downregulation of CD86, CD80, and MHC-II in TAM-MDMs, which decreased antigen presentation by TAM-MDMS and drove a more immunosuppressive tumor microenvironment [139]. Sex-related factors also influence the reprogramming of TAM-MDMs. Ochocka et al. reported elevated expression levels of MHC-II and PD-L1 within TAM-MDMs among male individuals, suggesting more tumor-supportive features of male myeloid cells than their female counterparts [127, 140].

#### Microglial reprogramming in the tumor microenvironment

Data suggest that when activated by glioma cells, microglia have reduced phagocytic potential compared to microglia in homeostasis [141–143]. These findings provide early proof that microglia are reprogrammed in the tumor microenvironment. However, even under normal conditions, microglia in different brain areas exhibit phenotypic differences. For example, aging strongly affects microglia in the cerebellum and hippocampus but not those in the cortex or striatum [60]. Such phenotypic differences might influence glioma progression. Indeed, compared with cortical microglia in normal brain samples, Lin et al. found that TAM-MGs isolated from grade IV diffuse midline glioma with H3K27M mutation expressed higher VEGFA and TGBFI levels [144]. The presence of development-associated microglial subpopulations might also affect tumor initiation and development. For instance, CD11c+ microglia that mainly exist during the early postnatal developmental stages exhibit robust *Igf1* gene expression, which is crucial for tumor growth in a murine model of medulloblastoma [28, 63, 145, 146].

New technologies, such as scRNA-seq combined with time-of-flight mass cytometry, have recently helped dissect microglial heterogeneity in the context of glioma [121]. Studies leveraging these approaches have shown that proinflammatory cytokine expression [121] and the response to type I interferons and hypoxia-associated molecules [121] are upregulated in microglial subpopulations in resected tumor tissue. Specifically, a proinflammatory subpopulation of microglia found in resected tumor tissues express high levels of *Il1b*, *Ifnb1*, *Ccl4*, *Il12*, and Tnf [126, 135, 147]. Others identified a subset of proinflammatory microglia that accumulated at the interface between the glioma lesion and normal brain tissue and highly expressed *Ccl3* [148]. Finally, proinflammatory microglia with increased expression of the complement cascade genes *C1qa*, *C1qb*, *C1qb* and *C4b* were observed in a mouse model of the YAP1 gene fusion subtype of pediatric ependymoma [149].

DAM-like microglial subsets may also be present in glioma [121]. The signature genes of this subpopulation include *Spp1*, *Gpnmb* and *Cst7* (Qian J, 2021, unpubl. data). Using RNAscope, we found that these cells are mainly located at the tumor periphery and infiltrate into the superficial area of the tumor lesion [138](Qian J, 2021, unpubl. data). These signatures are similar to those of DAMs found in Alzheimer’s disease and thus might reflect common phenotypic characteristics of microglia in response to pathological challenges.

Overall, the contributions of these various microglial subtypes to glioma development remain under speculation. Considering the high heterogeneity and plasticity of microglial populations, we posit that the microglial state is likely to be transitory and strongly context dependent.

#### Macrophages as prognostic markers for glioma

As discussed, TAMs, and more specifically MDMs, are associated with a poor prognosis in glioma. Indeed, TAM abundance in the tumor microenvironment negatively correlates with glioma prognosis, especially in IDH-wildtype and mesenchymal subtypes [147, 150–152]. Consistently, expression of the MDM signature gene *CD204* is associated with a shorter survival in affected patients [152]. Some data suggest that the negative correlation between TAM infiltration and survival only holds true for adult patients with malignant gliomas of the mesenchymal subtype [153], but contradictory data also show a positive correlation between CD68 + CD163 + CD206 + TAM infiltration and the overall survival of patients with IDH1R132H-WT GBM [154]. Karimi et al. applied imaging mass cytometry to characterize the immunological landscape of patients with GBM and identified a unique population of myeloperoxidase (MPO)-positive macrophages associated with long-term survival [155]. Based on these discrepancies, there may be different subpopulations of macrophages that exert antitumor or protumor effects in gliomas, and further accurate identification of different subpopulations may be required to more precisely study the prognostic value of different subpopulations of macrophages.

Sørensen et al. found that the abundance of Iba1+ microglia was significantly associated with the survival of glioma patients [152]. Finally, a recent study demonstrated the ability to detect TAM infiltration with an MRI radiomics approach [156], meaning that macrophages could be readily and continuously monitored in clinical settings via noninvasive detection approaches. Studies have now also mentioned the involvement of peripheral-derived macrophages in processes such as degenerative diseases and aging [24, 157]. By using methods such as MRI radiomics, it is also possible to follow macrophages in diseases that would be impossible to sample surgically, and further studies are needed to confirm the association of macrophages with these brain disorders.

These findings suggest that evaluating the infiltration of TAM-MDMs and TAM-MGs might serve as a potential prognostic or classification marker for glioma. A more precise definition of macrophage subpopulations may provide additional clinical cues for the future and could explain findings that are currently not entirely consistent. Moreover, emerging technologies, such as radiomics, might facilitate more specific diagnoses of glioma and risk stratification of affected patients. However, whether these cells have similar value in other CNS disorders remains to be confirmed.

#### Macrophages as therapeutic targets for glioma

TAMs, when considered as an entire population, generally promote tumor development, so their infiltration into the tumor microenvironment correlates with a worse prognosis for glioma patients. As such, these cells might be potential therapeutic targets, and great efforts have been made to either deplete these cells or prevent their infiltration. Methods such as CSF1R inhibition have been used to deplete TAMs [158, 159], or CCR2/CCL2 antibody treatment has been used to prevent TAM recruitment [160, 161]. These therapeutic regimens showed a good response in animal models, but significant results have not been reported in clinical trials. The outcomes of such therapies might be affected by the TAM infiltration level, the ratio of microglia to MDMs, the integrity of the BBB, and/or the individual tumor immune landscape. Considering the high plasticity and heterogeneity of TAMs, extensive elimination of all subpopulations might elicit other adverse effects. Efforts are therefore needed to precisely inhibit only tumor-promoting MDM subsets.

Other attempts to design effective therapeutics have aimed to use monocytes and MDMs as potential drug-delivery vehicles or so-called “Trojan cells” in glioma treatment [162]. This seems to be a promising approach considering that these cells can theoretically be loaded with any drugs to cross the BBB and precisely target the tumor lesion, avoiding affecting healthy brain tissue [163]. Nevertheless, there are also problems to be solved, such as the reduced bioactivity of drugs after digestion by cell lysosomes and the toxic effects of drug accumulation in the periphery if carrier cells do not manage to enter the tumor.

Clearly, the role of macrophages in glioma is complex, as different subpopulations can be beneficial or detrimental. Developing strategies to selectively target protumor cells while preserving antitumor cells is an area of active research in the field of glioma cancer therapy.

### Outlook

Many of the developments in understanding brain macrophage heterogeneity in development, health, aging and disease have come from scRNA-seq studies. Now, we need to understand the level of functionalization this heterogeneity brings. Studies must now focus on the molecular interactions between cells but also reorganize all the knowledge that has accumulated with a spatial dimension. As we enter a new era of spatial transcriptomics and proteomics, further advances in our understanding of the interactions of brain macrophages with other cells and their corresponding molecular programs that affect specific areas of the brain will also reveal novel therapeutic approaches for patients who are affected by developmental or age-related disorders, neurological diseases and cancers with a macrophage component.

Through this review, we have highlighted the impact of a loss of BBB integrity in neurodegenerative diseases and in cancer, and this BBB disruption allows the infiltration of monocytes and the emergence of new macrophage populations such as DIMs or TAM-MDMs in the brain. Enhancing our understanding of the functions of brain macrophage populations during development can reveal the cues that are necessary for the reemergence of specific programs in diseases, such as the DAM program compared to CD11c+ microglia/YAM.

We must also keep in mind the complex interplay among the brain, the gut microbiome, and bone marrow myelopoietic aging in the context of neurodegenerative diseases. This observation can be extended to glioma, as it is likely that while some macrophage programs observed in pediatric cancer could also be observed in adult cancer, some programs could be unique to adulthood or childhood. A better understanding of how macrophage heterogeneity functionally impacts the neuronal and glial environment will be key to allowing us to develop innovative therapeutics for neurodegenerative diseases and brain glioma.
